# Laparoscopic management of appendicular mucinous cystadenoma, case report

**DOI:** 10.1016/j.ijscr.2018.11.068

**Published:** 2018-12-12

**Authors:** Mohammed S. Foula, Abdullah M. Alardhi, Sharifah A. Othman, M. Khalid Mirza Gari

**Affiliations:** a Department of Surgery, King Fahad University Hopsital, Imam Abdulrahman Bin Faisal University, Khobar, Saudi Arabia; b Imam Abdulrahman Bin Faisal University, Dammam, Saudi Arabia

**Keywords:** Mucinous cystadenoma, Appendicular mucocele, Laparoscopy

## Abstract

**Introduction:**

Appendectomy is the most common emergency surgical procedure performed worldwide. Mucinous cystadenoma is a rare benign tumor of the appendix. There is no agreement on the best surgical approach for its management. Recently, laparoscopic approach is being increasingly tried. Careful excision of the tumor is mandatory to avoid content spillage into peritoneum resulting in pseudomyxoma peritonei.

**Case presentation:**

A middle-age male patient presented to the emergency department complaining of chronic abdominal pain, bleeding per rectum and recurrent attacks of vomiting. Preoperative imaging confirmed presence of cystic lesion in the right lower quadrant. He underwent a diagnostic laparoscopy with resection of appendicular mucocele. The histopathological examination confirmed the diagnosis of appendicular mucinous cystadenoma. He was followed up in the clinic for two years.

**Conclusion:**

Appendicular mucinous cystadenoma should be considered in differential diagnosis of cystic mass detected in the right lower quadrant of the abdomen on US or CT. Laparoscopic excision of the tumor is safe and feasible with extra care taken to avoid pseudomyxoma peritonei.’

## Highlights

• Appendicular mucinous cystadenoma is a rare benign tumour of the appendix.
• It should be considered in the differential diagnosis of cystic lesion in right lower quadrant of the abdomen.
• Care must be taken during the operation not to cause spillage of its content.
• Laparoscopic surgery in patients with appendicular mucinous cystadenoma is technically challenging but can be done safely by experienced laparoscopic surgeon.

## 1 Introduction

Appendectomy is the most common emergency surgical procedure performed worldwide [1]. Acute appendicitis is the main histopathological diagnosis, however, many other appendicular diseases may be the cause [2]. Mucinous cystadenoma is a rare benign tumor of the appendix with an incidence rate of 0.6% of all appendectomy specimens [3]. If diagnosed preoperatively, available surgical options are appendectomy or right hemicolectomy with no agreement on the best surgical approach. Recently, laparoscopic approach is being increasingly tried. Careful excision of the tumor is mandatory to avoid content spillage into peritoneum resulting in pseudomyxoma peritonei.

We report a case of young male with appendicular cystadenoma with successful laparoscopic management. This work is reported in line with SCARE criteria [4].

## 2 Case report

A 37-year-old male patient presented to the emergency department complaining of three days history of abdominal pain, bleeding per rectum, nausea and recurrent attacks of vomiting. The pain was recurrent for the past three months and increased over the last month. On examination, the patient was malnourished and pale. He was vitally stable. His abdomen was soft, lax, without evidence of peritonitis. No masses could be appreciated. Digital rectal examination revealed blood on the glove with no masses or hemorrhoids. Routine blood tests were within normal ranges.

**Fig. 1:**
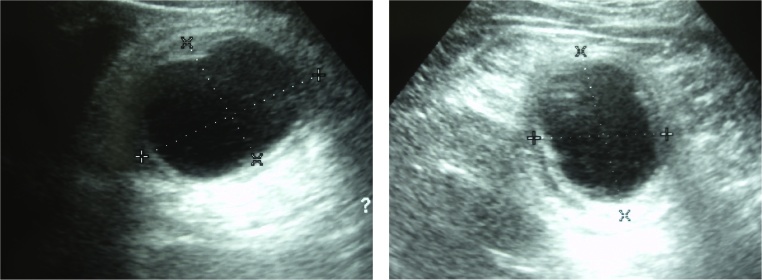
Abdominal ultrasonography showing an oval-shaped hypoechoic lesion in the right lower quadrant area.

**Fig. 2:**
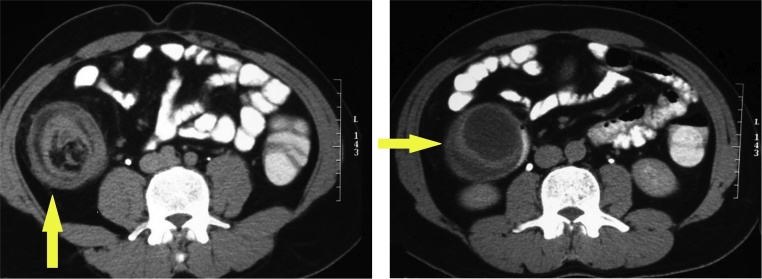
Contrast-enhanced abdominal computed tomography CT showing a cystic lesion within the lumen of cecum with thick septations (yellow arrow) (For interpretation of the references to colour in this figure legend, the reader is referred to the web version of this article).

Abdominal ultrasonography US showed a well-defined oval-shaped hypoechoic lesion in the right lower quadrant area (Fig. 1). Contrast-enhanced abdominal computed tomography CT showed a well-defined cystic lesion within the lumen of cecum with thick septations measuring 4 × 4 cm (Fig. 2). As well, a doughnut shape was seen suspecting ileocecal intussusception. No enlarged or suspicious lymph nodes were detected in CT. As well, no free intraperitoneal fluid was seen. Colonoscopy revealed a cystic swelling in the cecal submucosa occupying half of its circumference. Biopsy from the mass was technically difficult. Advancement of the scope was impossible due to obstruction of the ileocecal valve by the mass.

The patient was kept nil per mouth and on intravenous fluid. He underwent elective diagnostic laparoscopy. He was placed in supine, Trendelenburg position. Closed pneumoperitoneum was created using a Veress needle in Palmer’s point. The scope was inserted through a supra-umbilical incision using an 11-mm Visi-port trocar. Two trocars were inserted five centimeters below the costal margins at right and left midclavicular lines. Diagnostic laparoscopy showed a mass involving the appendix, the ileocecal junction and the cecum with no free fluid in the peritoneal cavity. Devascularization was done starting five centimeters proximal to the ileocecal junction up to the hepatic flexure. Transection of distal ileum and transverse colon distal to hepatic flexure was done using Endo-GIA tristapler. Side-to-side ileo-transverse anastomosis was created using Endo-GIA tristapler. The specimen was retrieved en-bloc using Endo-bag. The patient tolerated the procedure well. He started clear liquid on the third postoperative day. He was discharged home on the sixth postoperative day.

Histopathological examination of the specimen showed mucinous cystadenoma of the appendix with extravasation of mucinous material into the submucosa of the cecum, leading to formation of a pseudocyst. No malignant cells were found in the resected ileocolic lymph nodes. All margins were free from malignant cells. After multidisciplinary meeting with medical oncology, pathology and radiology teams, there was no need for any further surgical intervention nor follow-up imaging. He was followed up regularly in the surgical clinic for two years with no symptoms or signs.

## 3 Discussion

Despite its rarity, mucinous cystadenoma of the appendix is the most common appendicular benign neoplasm representing 0.6% of all appendectomy specimens [3]. It is one cause of appendicular mucocele which is abnormal accumulation of mucoid material inside the appendicular lumen. Other causes of appendicular mucocele include retention cyst, mucosal hyperplasia or mucinous cystadenocarcinoma. Classically, it presents as acute appendicitis, discovered intraoperatively and confirmed by histopathological examination of the specimen. Also, it may present as chronic abdominal pain, bleeding per rectum abdominal mass or intussusception [5].

Its preoperative diagnosis is usually challenging. Ultrasonography of the abdomen may show a well-encapsulated cystic lesion containing onion skin-like layers with variable echogenicity. CT scan is superior where it appears as a well-encapsulated cystic lesion with variable wall thickness. Colonoscopy is mandatory in case of appendicular mucinous cystadenoma to rule out associated colon neoplasms which is reported in 20% of cases. Likewise, few articles in the literature reported appendicular mucinous cystadenoma coexisting with appendicular carcinoid tumors [6].

There is no consensus in the literature regarding the best surgical approach to deal with appendicular mucocele [7]. Laparotomy is the standard approach and recommended by many authors as it ensures avoidance of mucocele rupture and seeding of trocar sites [8]. Care must be taken intraoperatively, especially if laparoscopic approach is adopted, not to cause content spillage leading to formation of pseudomyxoma peritonei. Right hemicolectomy is indicated if cecum or appendicular base is involved [5,9]. Only few case reports have discussed successful management of appendicular mucocele laparoscopically [10].

Further management depends on 1- integrity of the mucocele, 2- involvement of the base of the appendix, 3- the peritoneal fluid cytology: positive or negative, and 4- the regional lymph nodes: positive or negative. No long-term follow-up is needed in case of intact mucocele with negative cytology, negative lymph node and negative margins of resection [8].

In our case, the patient presented by chronic abdominal pain and bleeding per rectum. The preoperative imaging and colonoscopy confirmed presence of cecal cystic lesion. We preferred to start with laparoscopic technique with minimal threshold to convert to open laparotomy. The patient was planned for elective diagnostic laparoscopy and possible right hemicolectomy. Fortunately, the case was totally managed laparoscopically. To avoid spillage of its contents, dissection was carried out using atraumatic intestinal clamps without direct manipulation of the appendix or the mass. As well, the whole specimen was removed via Endo-bag. The histopathological examination was definitely diagnostic.

## 4 Conclusion

Appendicular mucinous cystadenoma should be considered in differential diagnosis of cystic mass detected in the right lower quadrant of the abdomen on US or CT. Laparoscopic excision of the tumor is safe and feasible with extra care taken to avoid pseudomyxoma peritonei.

## Conflict of interests

No conflict of interests.

## Funding

No funds or sponsors.

## Ethical approval

Case reports are exempted from ethical approval.

## Consent

Witten informed consent was obtained from the patient for publication of this case report.

## Author contribution

Dr. Mohammed S. Foula: main author, writing the paper, reviewing article

Dr. Abdullah M. Alardhi: data collection, writing the paper

Dr. Sharifah A. Othman: data collection

Dr. M Khalid Mirza Gari: study concept, reviewing article, supervisor

## Registration of research studies

None.

## Guarantor

Mohammed S. Foula.

## Provenance and peer review

Not commissioned, externally peer-reviewed.
